# Changes of Peripheral Nerve Function and Vitamin B_12_ Level in People With Parkinson's Disease

**DOI:** 10.3389/fneur.2020.549159

**Published:** 2020-10-29

**Authors:** Feng Qiu, Yue Wu, Hui Cao, Ben Liu, Mingyang Du, Haibo Jiang, Shun Li

**Affiliations:** ^1^Cerebrovascular Disease Center, Nanjing Brain Hospital Affiliated to Nanjing Medical University, Nanjing, China; ^2^Neonatal Medical Center, Children's Hospital of Nanjing Medical University, Nanjing, China; ^3^Department of Physical Diagnosis, Nanjing Brain Hospital Affiliated to Nanjing Medical University, Nanjing, China

**Keywords:** peripheral nerve, sensory, vitamin B_12_, balance, Parkinson's disease

## Abstract

**Background and Purpose:** Peripheral nerve function plays an important role in balance control. Impairment of peripheral sensory information appears in people with Parkinson's disease (PD). Furthermore, there is a link between peripheral nerve disorders and vitamin B_12_ level. Here, we studied whether there were deficits of peripheral nerve function and vitamin B_12_ level, which may lead to decreased postural stability in PD.

**Methods:** Fifty PD and 50 age-matched healthy subjects were enrolled in the study. This study evaluated folic acid and vitamin B_12_ levels in serum. Postural balance was studied according to the clinical Tinetti scale. Some comprehensive physiological assessments of peripheral nerve functions, including peripheral sensation, the perception of temperature, pain, and touch sensations, were also undertaken in this study.

**Results:** Compared with the control group, vitamin B_12_ and folic acid were decreased in PD (*P* < 0.05). Furthermore, the PD group exhibited declines in peripheral nerve functions, including touch, temperature, pain, and nerve conduction velocity (*P* < 0.05). Statistical tests identified a significant association between decreased peripheral nerve function and poor balance according to the Tinetti scale (*P* < 0.05). Low vitamin B_12_ levels were also associated with deficits of peripheral nerve function, cumulative levodopa dose, and poor balance in PD (*P* < 0.05).

**Conclusions:** Data suggested that peripheral nerve function was impaired in people with PD. Deficits of sensory input and low vitamin B_12_ level may contribute to balance deficits in PD.

## Introduction

Parkinson's disease (PD) is a neurodegenerative disease, which manifests due to the loss of dopamine cells in the substantia nigra area. Although it is argued that the most important motor dysfunction in PD is bradykinesia, other motor dysfunctions, such as resting tremor, increased muscle rigidity, and decreased postural reflex are also classic symptoms in PD (1). Traditionally, motor deficits are considered to be the key factors affecting postural stability in patients living with this chronic and progressive brain disease. However, impairments of peripheral sensory function may also occur in people with PD and impact the patients' ability to maintain equilibrium. Little has been done to look into the nature and extent of sensory impairments in people with PD. Recently, researchers have started focusing on the effects of impaired peripheral nerve function on a PD patient's ability to adjust postural balance (2–4). Compared with normal subjects, decline of peripheral sensory information is more common in PD individuals. Given peripheral sensory information plays such an essential part in postural balance, research seeking to provide insight into the influence of peripheral nerve function changes in PD patients is desperately needed.

Furthermore, previous reviews (5, 6) have described a link between peripheral nerve disorders and vitamin B_12_ level, indicating that poor nerve function was associated with decreased vitamin B_12_ level. The peripheral neuropathy resulting from vitamin B_12_ deficiency may lead to deficits of postural balance in people with PD. However, few studies have focused on such associations in PD. Therefore, there is an urgent demand on evaluating the associations between peripheral nerve functions and vitamin B_12_ level in people with PD. This study aimed to evaluate the differences of peripheral nerve function and serum biochemical indexes between people with PD and healthy individuals by using a series of physiological assessments.

## Method

All experimental procedures were approved by the Human Research Ethics Committee of Nanjing Brain Hospital, affiliated to Nanjing Medical University.

### Subjects

Fifty people with PD were enrolled in the current research. All subjects were required to be clinically diagnosed with PD and were assessed while taking their regular anti-parkinsonian medications. Levodopa equivalent daily dose and cumulative levodopa dose were calculated for each PD patient (7). Severity of PD symptoms was assessed using the Unified Parkinson's Disease Rating Scale (UPDRS) (8) and the Hoehn and Yahr Stage Score (9). Another 50 healthy individuals were also enrolled to serve as controls for the PD patients. Subjects in both the PD and control groups were free of dementia, tumor, diabetes, arthritis, unstable heart disease, or other acute diseases. Furthermore, PD patients and control subjects were ruled out if they had heavy alcohol use and were required to be without vitamin B_12_ treatment for the 4 weeks preceding data collection and were required to not be routinely taking any folic acid or multivitamin supplements.

### Experimental Protocol

Serum levels of folic acid and vitamin B_12_ were tested in all subjects using methods outlined later, whereas balance was studied according to the clinical Tinetti scale (10). Comprehensive physiological assessments of peripheral sensory functions were conducted, which included test of peripheral sensation, the perception of temperature, pain, and touch.

### Folic Acid and Vitamin B_12_ Level

Fasting venous blood (4 ml per person) was obtained from all subjects in the morning. The serum was then separated by centrifugation in the Hospital's biochemistry laboratory to allow vitamin B_12_ and folic acid levels to be assessed. Specifically, vitamin B_12_ was measured using a microparticle enzyme immunoassay performed using the AxSYM automatic immune analyzer (AxSYM system; Abbot Laboratories, USA) (11). In contrast, serum folic acid levels were measured using an ELISA kit (Wes-Tang Bio-Tech, Shanghai, China). Absorbances (450 nm) were read using a microplate reader (12). In accordance with Nanjing Brain Hospital's standards, normal range of vitamin B_12_ was defined as being between 189 and 883 pg/ml, whereas the normal range of folic acid was defined as being between 1.1 and 20.5 ng/ml.

### Touch Sensation

**Figure 1:**
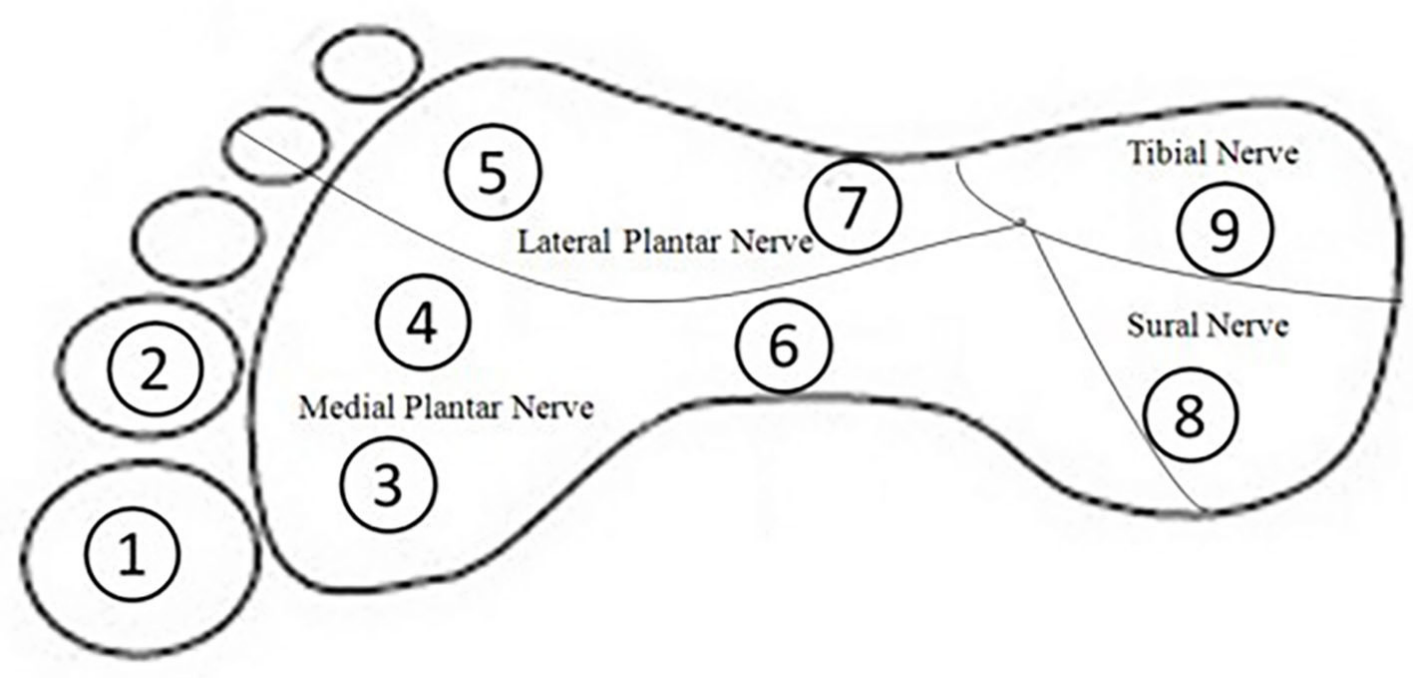
Foot stimulation positions evaluated by Semmes–Weinstein-type pressure aesthesiometer.

To assess touch sensitivity of the ankle and plantar surface of the foot, Semmes–Weinstein monofilament pressure aesthesiometers (North Coast Medical, California) were used. Altogether, 10 positions were chosen, including (1) the lateral malleolus of the ankle, which has been evaluated extensively in previous studies (13, 14); and (2) nine positions located on the plantar surface of the foot, which were chosen according to previous studies (15). These nine test positions represented areas innervated by four main peripheral nerves: position 1, 2, 3, 4, and 6 for medial plantar nerve, positions 5 and 7 for lateral plantar nerve, position 8 for tibial nerve, and position 9 for sural nerve, respectively (16) (Figure 1).

### Proprioception

**Figure 2:**
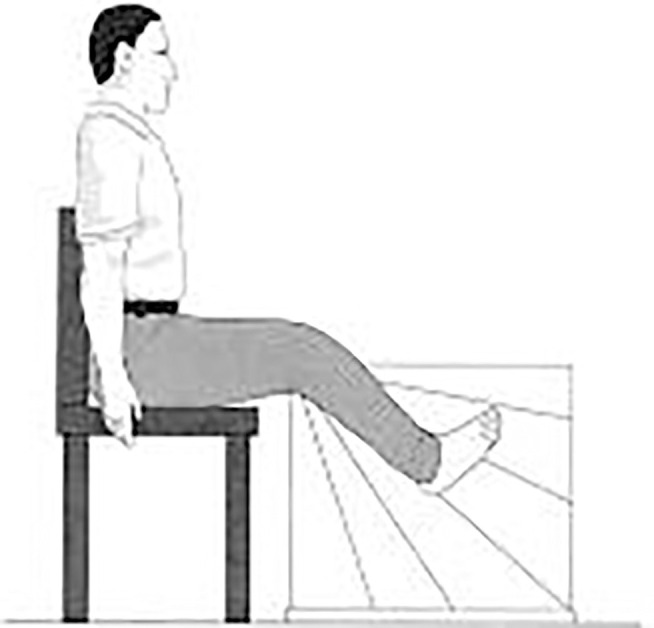
Subject matching the feet on the acrylic sheet during the proprioception test (13).

Lower-limb proprioception (13), an important part of peripheral sensation, was also evaluated during sitting with eyes closed. Subjects were seated on a high stool with a thick clear acrylic sheet positioned between their feet. The vertical surface of the acrylic sheet was inscribed with a protractor that showed angular increments of 2° (Figure 2). A small cross was marked on the medial aspect of the first metatarsal head on both feet. Then subjects were required to match their legs in five trials on either side of the plastic board while blindfolded. During each trial, one lower limb was rotated and the foot was placed against the side of the plastic board by the experimenter. Participants were then required to match this position with their other leg by actively moving it and placing it against the side of the plastic board. Within each trail, three positions on the board were chosen randomly for each foot: (1) high position with knee angle at ~160°; (2) medium position with knee angle at ~130°; (3) low position with knee angle at ~100°. Errors in matching the lower limb were recorded in terms of degrees.

### Quantitative Thermal Sensory/Perception of Temperature and Nociception

Temperature and nociception (pain) of the feet were performed by using the Thermal Sensory Analyzer TSA-2001 (Medoc, Israel). The dorsum of the dominant foot was chosen as the test position. A thermode (9 cm^2^ contact probe) that can heat or cool the skin, as needed, was placed on the subject's skin at the starting temperature of 32°C. During the process, the subject can perceive changes of stimulations with the temperature increased or decreased continuously at a rate of 1°C per second (17). Four sensory sub-modalities were tested: warm sensation (WS), cold sensation (CS), heat-induced pain (HP), and cold-induced pain (CP). Each subject completed six trials (three WS and three CS trials) in a random order. In the process of induced pain assessments, subjects were required to respond by pressing the stop button once the thermode reached a temperature that was too hot or too cold for them to comfortably tolerate.

### Peripheral Nerve Electrodiagnosis

Peripheral motor and sensory nerve functions were evaluated using standard surface electroneurography (Natus Medical, Middleton, WI, USA). For these assessments, three main nerves (peroneal nerve, tibial nerve, and sural nerve) of the dominant leg were chosen. Measurement parameters included nerve conduction velocity, F-wave latency, amplitudes of muscle action potential, and sensory nerve action potential.

### Postural Stability Assessment

The Tinetti Balance and Gait assessment (10) was used to determine postural stability in older subjects. Risk of falls was evaluated during both gait and balance portions, with a maximum 12 points for gait score and 16 points for balance score (overall score is 28 points). Higher scores are considered to represent better postural stability.

### Statistical Analysis

Differences in peripheral nerve function within two groups were determined by using Student's *t*-test. As for the accuracy of temperature and pain detection, the χ^2^ test was used for the two groups. Data obtained were shown as mean ± SD values, whereas Pearson's and Spearman's rank correlations were used for correlation analysis. Statistical analyses were completed using the SPSS (version 21.0, Chicago, IL, USA) and a *p* < 0.05 was considered as statistically significant.

## Results

A total of 50 people (33 males and 17 females; mean age 66.4 ± 8.0 years) with PD and 50 controls (33 males and 17 females; mean age 64 ± 4.8 years) completed all assessments. No significant differences were observed in the demographic characteristics of both groups (Table 1). Based on the results of the clinical tests of disease severity, the PD patients were predominantly at an early stage of disease progression (Hoehn and Yahr score 1.5 ± 1.0) and had mild to moderate symptoms (UPDRS score = 38.7 ± 15.7). Among the 50 PD patients, only 6 patients were on carbidopa and 3 patients were on catechol-*O*-methyltransferase inhibitors. Levodopa equivalent daily dose and cumulative levodopa dose were calculated according to a previous study (7) (Table 2).

**Table 1:** Demographic characteristics of both groups.

	**Control (*n* = 50)**	**PD (*n* = 50)**	***P*-value**
Age (years)	64.0 ± 4.8	66.4 ± 8.0	0.078
Gender (male %)	66%	66%	
Height (cm)	166.4 ± 10.3	166.1 ± 8.9	0.722
Weight (kg)	67.6 ± 15.3	63.4 ± 12.7	0.091
BMI (kg/m^2^)	24.4 ± 3.9	23.0 ± 4.1	0.073

*Data are mean ± SD values*.

*BMI, body mass index*.

**Table 2:** Disease characteristics of Parkinson's patients.

UPDRS I	2.6 (2.0)
UPDRS II	11.7 (5.2)
UPDRS III	27.2 (9.5)
UPDRS Total	38.7 (15.7)
Hoehn and Yahr	1.5 (1.0)
Levodopa equivalent daily dose (mg)	380.34 (164.34)
Cumulative levodopa dose (g)	504.75 (411.92)

*Data are mean ± SD values*.

*UPDRS, Unified Parkinson's Disease Rating Scale*.

### Folic Acid and Vitamin B_12_ Level

Vitamin B_12_ and folic acid levels for both groups are shown in Table 3. People with PD were more likely to have low folic acid (*P* < 0.05). Similarly, the PD group also returned significantly lower levels of serum vitamin B_12_ (*P* < 0.01). However, both folic acid and vitamin B_12_ levels were within the normal range for all subjects.

**Table 3:** Levels of serum vitamin B_12_ and folic acid.

	**Control (*n* = 50)**	**PD (*n* = 50)**	***P*-value**
Folic acid (ng/ml)	12.74 ± 5.35	10.11 ± 4.28	0.041
Vitamin B_12_ (pg/ml)	553.61 ± 208.33	412.07 ± 154.09	0.009

*Data are mean ± SD values*.

### Touch and Proprioception

Compared with the controls, the PD patients had significant declines on touch thresholds at all 10 sites around the foot and ankle areas, resulting in poorer tactile sensitivity scores (4.3 ± 1.5 vs. 2.5 ± 0.6, *P* < 0.01). Despite the differences shown in tactile sensitivity, with respect to lower limb proprioception, no significant differences were noticed between two groups (*P* > 0.05) (Table 4).

**Table 4:** Touch sensation and lower limb proprioception in both groups.

	**Control (*n* = 50)**	**PD (*n* = 50)**	***P*-value**
**Touch sensation**			
Total score	2.5 ± 0.6	4.3 ± 1.5	<0.001
Ankle	2.6 ± 1.0	4.3 ± 1.6	0.002
Site 1	2.4 ± 1.2	4.1 ± 1.3	0.008
Site 2	2.2 ± 0.8	4.0 ± 1.8	0.002
Site 3	2.1 ± 0.9	4.6 ± 1.5	<0.001
Site 4	2.4 ± 0.5	3.9 ± 2.0	0.011
Site 5	2.5 ± 0.6	3.8 ± 1.7	0.009
Site 6	1.9 ± 0.7	3.1 ± 1.4	0.002
Site 7	2.1 ± 0.5	3.5 ± 1.4	0.006
Site 8	3.2 ± 1.0	5.6 ± 1.2	<0.001
Site 9	3.3 ± 0.8	5.7 ± 1.5	<0.001
Lower limb proprioception	3.9 ± 1.2	3.7 ± 1.3	0.523

*Data are mean ± SD values*.

*PD, Parkinson disease*.

### Thermal Perception

In addition to impaired touch sensation, the PD group also showed reduced capacity to detect temperature changes when compared with the control group. During both the trials involving a reduction in thermode temperature (28.2 vs. 21.3°C) and an increase in thermode temperature (40.2 vs. 43.1°C), the control group consistently identified the changes in temperature significantly sooner than the PD group (*P* < 0.05). Accuracy of response on temperature changes was also evaluated in this study during all tests (3 trials per subject), showing that there were 21 trials where PD patients could not sense an increasing change in temperature assessments (getting hotter) and 11 where PD patients could not sense a decreasing change in temperature (getting colder). Unlike the results from cold and warm temperature sensation assessments, the cold- and hot-induced pain assessments showed no differences between the two groups (*P* > 0.05). During the temperature-induced pain assessments, there were 22 trials where the PD patients did not sense painful stimuli in response to an increase in temperature (getting hotter) and 17 where PD patients did not sense pain in response to a decrease in temperature (getting colder). However, most subjects in the control group reported experiencing discomfort in the process of the cold- and hot-induced pain assessments (Table 5).

**Table 5:** Temperature and pain sensations in both groups.

	**Control (*n* = 50)**	**PD (*n* = 50)**	***P*-value**
**Temperature**			
Cold, degrees	28.2 ± 3.0	21.3 ± 12.5	0.013
Cold accuracy			<0.001
True	120	86	
False	29	43	
N/A	1	21	
Warm, degrees	40.2 ± 3.1	43.1 ± 4.2	0.038
Warm accuracy			0.009
True	141	124	
False	9	15	
N/A	0	11	
Pain			
Cold pain, degree	18.3 ± 6.6	14.2 ± 12.3	0.072
Cold pain accuracy			<0.001
True	112	70	
False	35	58	
N/A	3	22	
Hot pain, degree	47.3 ± 1.9	48.4 ± 2.5	0.482
Hot pain accuracy			<0.001
True	137	102	
False	13	31	
N/A	0	17	

*Data are mean ± SD values*.

*PD, Parkinson disease*.

### Nerve Conduction

Concerning motor nerve conduction velocity, subjects in the control group demonstrated faster velocity than the PD group for the tibial and peroneal nerve (both ankle–fibular head and fibular head–popliteal fossa sites) (*P* < 0.05). Compared with control group, significant declines of conduction velocity were recorded for the sural nerve in the PD group (*P* < 0.01). As for the sensory nerve, PD patients demonstrated significantly delayed latency of onset, compared with controls (*P* < 0.05), whereas 6% of controls and 34% of PD patients exhibited no nerve response at all. No significant differences on amplitudes of action potential were noticed for the three nerves between controls and PD patients (*P* > 0.05).

With respect to the F-wave analyses, 94% of controls and 86% of PD patients recorded an F-wave for the peroneal nerve. Similarly, only 88% of the PD patients displayed F-waves for the tibial nerve, whereas 96% of the control group exhibited this neurophysiological response. F-waves were observed for both groups during 10 repeated stimulations of the peroneal and tibial nerves; however, they occurred significantly more often in the control group (*P* < 0.05). F-wave latency was not significantly different between the two groups (*P* > 0.05) (Table 6).

**Table 6:** Electrodiagnosis results of two groups.

	**Control (*n* = 50)**	**PD (*n* = 50)**	***P*-value**
**Peroneal nerve**			
Motor NCV (AFH), m/s	47.5 ± 3.7	41.2 ± 4.6	0.037
Motor NCV (FP), m/s	48.1 ± 5.2	42.7 ± 7.3	0.015
M-wave AMP (A), mV	5.1 ± 1.8	4.7 ± 1.5	0.582
M-wave AMP (FH), mV	4.7 ± 1.5	4.6 ± 1.2	0.621
M-wave AMP (PF), mV	4.8 ± 1.3	4.9 ± 1.4	0.513
F-wave latency (A), ms	50.7 ± 3.1 (*n* = 47)	51.0 ± 2.3 (*n* = 43)	0.872
**Tibial nerve**			
Motor NCV, m/s	42.6 ± 4.1	39.7 ± 3.2	0.019
M-wave AMP (A), mV	6.5 ± 2.2	6.4 ± 1.7	0.742
M-wave AMP (PF), mV	5.8 ± 2.4	5.6 ± 2.5	0.305
F-wave latency (A), ms	51.7 ± 2.6 (*n* = 48)	53.2 ± 3.1 (*n* = 44)	0.227
**Sural nerve**			
Sensory NCV, m/s	53.2 ± 3.1 (*n* = 47)	48.9 ± 5.6 (*n* = 33)	0.011
Latency to onset, ms	3.2 ± 0.2 (*n* = 47)	3.5 ± 0.3 (*n* = 33)	0.013
SNAP AMP, μV	3.8 ± 2.2 (*n* = 47)	3.7 ± 1.7 (*n* = 33)	0.372

*Data are mean ± SD values*.

*NCV, nerve conduction velocity; A, ankle; AFH, ankle fibular head; FP, fibular head–popliteal fossa; FH, fibular head; PF, popliteal fossa; AMP, amplitude; SNAP, Sensory Nerve Action Potential; PD, Parkinson's disease*.

### Postural Stability Assessment

For the clinical tests of balance, the Tinetti Balance and Gait assessment showed that PD had reduced postural stability compared with the healthy individuals during both standing and walking (*P* < 0.05) (Table 7).

**Table 7:** Tinetti Balance and Gait assessment.

**Tinetti**	**Control (*n* = 50)**	**PD (*n* = 50)**	***P*-value**
Balance	15.8 ± 0.4	13.5 ± 2.2	0.004
Gait	10.5 ± 1.0	9.3 ± 1.7	0.022
Total	26.3 ± 1.3	22.8 ± 3.8	0.007

*Data are mean ± SD values*.

*PD, Parkinson's disease*.

### Correlations

In the PD group, there was a significant negative association between cumulative levodopa dose and vitamin B_12_ level (*R* = −0.831, *P* < 0.001). Peripheral nerve function demonstrated similar correlations, showing that cumulative levodopa dose was negatively associated with conduction velocity in both the tibial (*R* = −0.702, *P* = 0.003) and peroneal nerves (*R* = −0.737, *P* = 0.008). No significant correlations were observed between levodopa equivalent daily dose with other parameters (*P* > 0.05).

Correlation between peripheral nerve function and clinical balance scores demonstrated that there were negative associations between peripheral sensory (touch thresholds) and Tinetti clinical balance assessments (scores on gait: *R* = −0.669, *P* = 0.012, balance *R* = −0.831, *P* = 0.003 and total *R* = −0.711, *P* = 0.005) in the PD group.

As for temperature assessments, higher scores on clinical Tinetti gait and total tests were closely correlated with better cold sensitivities in PD patients (*R* = 0.541, *P* = 0.041; *R* = 0.601, *P* = 0.035). For pain assessments, increased thresholds were related to decreased scores in balance assessments in the PD group (*R* = −0.613, *P* = 0.022), whereas no significant correlations were observed in the controls.

Results on the nerve function tests revealed that Tinetti gait and total scores increased with the conduction velocity in both tibial and peroneal nerves (gait and tibial: *R* = 0.771, *P* = 0.008; gait and peroneal: *R* = 0.803, *P* = 0.002; total and tibial: *R* = 0.674, *P* = 0.014; total and peroneal: *R* = 0.708, *P* = 0.011). Furthermore, peroneal and tibial (ankle–fibular head site) nerve velocities were positively associated with the clinical Tinetti balance scores (*R* = 0.660, *P* = 0.032). No significant correlations were noticed in the control group.

As for serum tests, there were positive correlations between vitamin B_12_ and peroneal nerve velocity (*R* = 0.841, *P* = 0.002) in PD. Similar positive associations were noticed in vitamin B_12_ and Tinetti balance scores (*R* = 0.692, *P* = 0.018) for PD patients.

## Discussion

This current study investigated whether there were differences in peripheral nerve functions and serum vitamin B_12_ levels between PD and control. Whether these changes were associated with balance was also examined. Overall, significant decline in peripheral nerve function was observed in PD and significant positive correlations were also noticed between peripheral nerve function and postural stability. Furthermore, low vitamin B_12_ levels were associated with deficits of peripheral nerve function and poor balance in PD.

According to the results, it is consistent with previous studies (3, 18) that touch thresholds were increased and perceptions of sensory stimuli were reduced in the PD patients. This study revealed that all stimulation sites around the foot and ankle area demonstrated increased touch thresholds. Specifically, these positions corresponded to four main nerve distributions: lateral plantar, medial plantar, tibial, and sural nerve. Previous research (19) also pointed out that the plantar surface of the foot was spread with both slow and fast adapting cutaneous mechanoreceptors. According to monofilament stimulation, results showed that rapidly adapting receptors accounted for the main part, reaching almost 70% of the skin receptors. As for the PD group, these receptors were likely to be affected preferentially (20). It was noticed that impairments of peripheral sensation occurred in the people with PD; this finding was similar to results from other research that PD patients suffered from diminished proprioception and touch discrimination on the sole (3, 18). Furthermore, Nolano et al. (20) performed a series of skin biopsies for the lower extremities in PD, indicating that there was impaired peripheral sensory and cutaneous denervation. In our study, there was no significant decline in proprioception in people with PD, whereas other studies showed opposite results (3, 21). These contrasting results may be explained by differences in the limbs chosen to assess proprioceptive changes in people with PD. For example, most previous assessments of proprioception in PD patients have assessed the upper limb, whereas the current study reports on lower limb proprioception. It is well-known that the encoding of sensory information destined for the proprioceptive cortex is reliant on muscle spindles, Golgi tendon organs, and joint and cutaneous receptors. However, the upper and lower limbs are characterized by differences in muscle length, nerve conduction velocities, and pathway lengths between sensory receptor sites and the central nervous system (22). Furthermore, given the relatively greater importance of upper limb involvement in the performance of day-to-day tasks, the joints of the upper limb (e.g., elbows, wrists) may be more highly innervated and, hence, capable of sensing a smaller degree of angular change compared with joints of the lower limb (e.g., knee, ankle). Such differences in muscle length, neural path length, and density of innervation may explain the proprioceptive difference reported in this study and highlight the need for specificity when assessing proprioceptive changes in PD patients (23, 24).

In the current study, sensation thresholds of both warm- and cold-induced pain were increased, and even more PD patients failed to respond before the temperature reached the maximum limit. Given this situation, these thermally induced nociceptive thresholds were calculated as conservative estimates according to the maximum values. Previous research provided support for this study, indicating that there were reductions in mechanical pain perception and an increase in thermal thresholds in people with PD (20). Thermal, fast pain, and mechanical stimulations were controlled by Aδ-fibers, whereas transmission of slow pain sensation was controlled by C-fibers. Free ends of nerves were lost in these two types of fibers in PD, leading to deficits in signal transmission through dorsal column–medial lemniscus and spinothalamic tract pathways. As a result, PD patients failed to receive sensory stimulus of temperature and pain. In summary, our findings showed that worse performances were noticed in PD than the control during the whole temperature and pain assessments, collectively indicating deficits in the temperature and nociception sensation in PD.

As for nerve conduction assessments on the tibial and peroneal nerves, both velocity and F-wave frequency were decreased significantly in the PD group. This result was similar to findings in Toth's study (25), indicating that motor conduction velocities were slower in PD patients compared with controls. Toth considered peripheral neuropathy in PD was due to levodopa exposure and high methylmalonic acid levels.

With respect to M-wave amplitudes in the current study, similar muscle activation was noticed for both PD and control groups for the lower limbs. Compared with Abbruzzese et al. (26) who evaluated the peripheral nerve functions on the upper limbs, we assessed electrophysiological functions of the lower limbs. Different positions assessed in the current study may contribute to the differences noticed in the previous study. Although the findings on peroneal and tibial nerves that were in accordance with Abbruzzese's research were the latent phases of F-waves, neither study showed significant differences between the two groups. However, there were fewer PD patients than healthy individuals whose F-wave values were recorded in the current study. When stimulations were transmitted to the spinal cord and back again in PD patients, the breakdown may occur in the pathway. Thus, it cannot be ruled out that motor nerve had deficits on transmission in PD patients.

As for sensory nerve function, the findings in the sural nerve agreed with Toth et al. (25) whose study presented decreases in the velocity and prolonged latency in the PD group. This may also be explained by the biopsy results from Nolano's study (20) that demonstrated impaired sensory receptor functioning in PD, indicating there were potential degeneration of sensory nerve fiber which caused declines in nerve conduction velocity in people with PD. Biopsy results also indicated that there was loss of myelin in peripheral nerve endings and swelled nerve axons in PD patients. These findings also supported the possible mechanism that degeneration in layers of protein and fat around the peripheral nerve axons caused declines in nerve conduction velocity in the PD group (27).

It was also revealed that PD patients performed poorer than healthy individuals according to clinical balance assessments. Tinetti assessments have been widely used in evaluating balance in the aged group including PD individuals (28, 29). Sensory information from the cutaneous mechanoreceptors of the soles were decreased, which in turn may lead to decreased postural stability (30, 31).

Only PD patients demonstrated associations between some of the measurements of peripheral nerve and the clinical balance scores. When the process of information transmission was interrupted, sensations may not be transferred to the brain normally, which in turn impaired the posture adjustment. Compared with healthy individuals, lower scores on the clinical Tinetti assessments were recorded in PD patients, which were closely associated with declines on peripheral nerve functions, touch, temperature, and nociception sensation. These impairments may negatively influence the patients' capacity to safely perform and transition between tasks of daily life (e.g., sitting to standing, walking to turning) and provide insight into the seemingly cautious behaviors of patients' in more complex environments (32).

In this study, there were positive correlations between vitamin B_12_ and peripheral nerve function and balance scores in PD. Previous research (33) indicated that decreases were evident in vitamin B_12_ and folic acid in PD patients, who presented with neuropathy syndromes like numbness, pain, and sensory deficits in lower extremities, proving that vitamin B_12_ deficiency affected thin-myelinated fibers or unmyelinated fibers (34). The decrease in methylation of myelin basic protein leads to myelin damage. Low vitamin B_12_ also can affect homocysteine metabolism, which plays an essential role in neural axon and Schwann cell. Thus, high homocysteine is associated with low vitamin B_12_, activating immuno-inflammatory responses, which in turn interferes with the metabolism of dopamine transporter in PD (35). It indicated that low vitamin B_12_ levels is a critical factor concerning decreased peripheral nerve functions and impaired balance in PD patients. The associations between cumulative levodopa dose and vitamin B_12_ level in the current study also indicated that vitamin B_12_ levels were decreased with increasing exposure to levodopa in the PD group. This may be explained by the fact that accumulated levodopa can lead to higher homocysteine, which may pose a potential risk of inducing neuropathy (36, 37). In this study, no correlations were noticed between levodopa equivalent daily dose with other parameters. These findings may have been because most patients in this study were in the early stages of PD; hence, levodopa intakes were still at relatively low daily levels. The possible vitamin B_12_ deficiency and neuropathy were not obvious until future accumulated. This finding was also in accordance with the results of this study, which indicated that vitamin B_12_ levels were within the normal range for all subjects, as were the nerve conduction assessments.

Although this study has, for the first time, assessed the peripheral neurological function of PD patients and correlated the nerve function with outcomes derived from common clinical assessment of balance, there are a few limitations to acknowledge. First, due to the clinical design, this study is limited by the lack of an assessment of methylmalonic acid levels, which may be associated with low vitamin B_12_. Similarly, given our methods, it is difficult to rule out the possibility that some participants presented with impaired glucose intolerance that was sufficient to cause neuropathy. Nevertheless, participants were screened for evidence of diabetes before their enrollment in the study, and all blood glucose levels were within the normal range. Future studies may seek to conduct an oral glucose tolerance test for all participants before their involvement to rule out its possible confounding effects. A further drawback is that we only assessed the dominant leg of each participant to maintain consistency between patients and controls. Finally, this study would have benefited from more patients and from the inclusion of biopsy data, both of which we plan to include in our future studies. These limitations may influence the nature of the results presented and should be noted when considering their implications to the wider PD population.

## Conclusion

To summarize, this study revealed that peripheral nerve function was impaired in people with PD. These deficits were noticed in touch sensation, temperature and nociception, and nerve functions. Deficits of sensory input and low vitamin B_12_ level may lead to declines in postural balance in PD. Given these deficits in sensory function, people with PD may benefit from the prescription of alternate therapies, such as exercise-based interventions (38, 39), to improve the patients' balance and reduce their falls risk.

## Data Availability Statement

The raw data supporting the conclusions of this article will be made available by the authors, without undue reservation.

## Ethics Statement

The studies involving human participants were reviewed and approved by Human Research Ethics Committee of Nanjing Brain Hospital, Affiliated to Nanjing Medical University. The patients/participants provided their written informed consent to participate in this study.

## Author Contributions

FQ and YW conceived the study, designed the experiments, analyzed the data, and wrote the article. HC provided the preprocessed data. BL and HC carried out experiments. MD, HJ, and SL helped to analyze the data and experiments result. All authors contributed to article revision, read, and approved the submitted version.

## Conflict of Interest

The authors declare that the research was conducted in the absence of any commercial or financial relationships that could be construed as a potential conflict of interest.
