# Corrigendum: Changes of Peripheral Nerve Function and Vitamin B_12_ Level in People With Parkinson's Disease

**DOI:** 10.3389/fneur.2020.643538

**Published:** 2021-01-28

**Authors:** Feng Qiu, Yue Wu, Hui Cao, Ben Liu, Mingyang Du, Haibo Jiang, Shun Li

**Affiliations:** ^1^Cerebrovascular Disease Center, Nanjing Brain Hospital Affiliated to Nanjing Medical University, Nanjing, China; ^2^Neonatal Medical Center, Children's Hospital of Nanjing Medical University, Nanjing, China; ^3^Department of Physical Diagnosis, Nanjing Brain Hospital Affiliated to Nanjing Medical University, Nanjing, China

**Keywords:** peripheral nerve, sensory, vitamin B_12_, balance, Parkinson's disease

In the original article, the reference for Marsh et al. (1) [reference (32) in the original paper] was incorrectly written as “Marsh R, Cole MH, Dissanayaka N, Au T, Clewett S. Ofile approach to falls risk assessment and prevention. *Phys Ther*. (2019) 83:237–52.”

It should be Marsh R, Cole MH, Dissanayaka NNW, Au TR, Clewett S, O'Sullivan JD, et al. The CuePed trial: how does environmental complexity impact cue effectiveness? A comparison of tonic and phasic visual cueing in simple and complex environments in a Parkinson's disease population with freezing of gait. *Parkinsons Dis*. (2019) 2019:2478980. doi: 10.1155/2019/2478980.

The authors apologize for this error and state that this does not change the scientific conclusions of the article in any way. The original article has been updated.
